# Vasoactive Hormones and the Diabetic Kidney

**DOI:** 10.1100/tsw.2008.57

**Published:** 2008-04-20

**Authors:** Christine Maric

**Affiliations:** Department of Medicine, Georgetown University Medical Center, Washington, DC, USA

**Keywords:** diabetes, diabetic nephropathy, kidney, renin-angiotensin-aldosterone system, endothelin, urotensin II, kallikrein-kinin system

## Abstract

Diabetic nephropathy is the single most common cause of end-stage renal disease (ESRD) and accounts for significant morbidity and mortality. While the incidence of ESRD increased dramatically in the 1980s and 1990s, the U.S. Renal Data System (USRDS) 2005 Annual Data Report shows that 338 out of every million Americans had kidney failure in 2003, down slightly from 340 per million in 2002. This report shows that the numbers of people developing ESRD have stabilized despite the persistent increase in the number of people diagnosed with type 2 diabetes mellitus (T2DM). These data attest to both the efficacy of the currently available therapeutic regimens for the treatment of ESRD as well as better overall patient care. Unfortunately, these encouraging statistics do not apply to all patients. According to the USRDS report, the most marked ESRD decrease was seen in young Caucasian men (<40 years of age), while in other patient groups, particularly African Americans, ESRD has not changed much at all. These observations suggest that more in-depth studies, addressing specific issues, such as race, are needed to understand the disease process fully in order to create novel therapeutic strategies to eradicate the disease completely in all patient populations.

The most commonly used therapeutic treatments for diabetic nephropathy are angiotensin-converting enzyme (ACE) inhibitors and angiotensin II (Ang II) receptor blockers (ARBs), implicating the importance of the renin-angiotensin-aldosterone system (RAAS) in the pathophysiology of diabetic nephropathy. The RAAS is not the only vasoactive hormonal system that is involved in the disease process. Over the past decade, studies have suggested that other vasoactive hormones, including endothelin, urotensin II, and the kallikrein-kinin system (KKS), are instrumental in mediating structural and functional alterations in the renal vasculature and parenchyma, leading to the development and progression of diabetic nephropathy. This review will summarize our current understanding of the contribution of vasoactive hormones in the pathophysiology of diabetic nephropathy with specific emphasis on the RAAS, especially the more recently identified components of this hormonal pathway.

## INTRODUCTION

Diabetic nephropathy is the leading cause of end-stage renal disease (ESRD) worldwide and is associated with increased cardiovascular risk, high morbidity, and mortality[1,2]. Despite the increasing prevalence of diabetes mellitus globally and the predictions that the prevalence of ESRD is likely to increase with the epidemic of diabetes, the most recent U.S. Renal Data System (USRDS) report suggests that the numbers of people developing ESRD due to diabetes have stabilized[3]. While this report seems to indicate that the currently available treatments for diabetic nephropathy are effective in attenuating the disease progression, the number of patients with ESRD is still very high and diabetic nephropathy remains a significant problem in terms of economic burden as well as quality of life.

Clinically, diabetic nephropathy is characterized by an initial increase in glomerular filtration rate (GFR) and microalbuminuria[4,5]. As a consequence of uncontrolled hyperglycemia, this stage of hyperfiltration coupled with glomerular hypertrophy is followed by proteinuria, glomerular and tubulointerstitial damage, accompanied by a decline in GFR and hypertension, ultimately leading to ESRD[6,7].

Diabetic nephropathy is generally believed to develop as a result of an interaction between multiple metabolic and hemodynamic factors, which activate common intracellular signaling pathways that, in turn, trigger the production of cytokines and growth factors, leading to renal disease. More specifically, prolonged and uncontrolled hyperglycemia that characterizes diabetes has been shown to activate nonenzymatic glycation and oxidative stress, polyol pathway, and hexosamine flux[8,9,10]. Persistent hyperglycemia also perturbs the renal hemodynamics through activation of a number of vasoactive hormonal pathways, including the renin-angiotensin-aldosterone system (RAAS), endothelin, and urotensin II[11,12,13,14,15]. These vasoactive hormones, in turn, activate common second messenger signaling pathways (such as protein kinase C [PKC] and MAP kinase [MAPK]), transcription factors (such as nuclear factor-κB [NF-κB]), which alter expression of genes of growth factors, and cytokines (such as transforming growth factor-beta [TGF-β], vascular endothelial growth factor [VEGF], platelet-derived growth factor [PDGF], and connective tissue growth factor [CTGF]), all of which have been implicated in the development of albuminuria, glomerulosclerosis, and tubulointerstitial fibrosis associated with diabetic nephropathy[16,17,18,19]. Fig. 1 depicts the cross-talk between metabolic and hemodynamic pathways that lead to the development and progression of diabetic nephropathy.

### Renin-Angiotensin-Aldosterone System (RAAS)

One of the best described and most researched vasoactive hormonal pathways in the pathophysiology of diabetic nephropathy is the RAAS. The RAAS has classically been thought to be part of the major regulatory system involved in blood pressure control as well as in water and electrolyte homeostasis[20,21]. However, studies in the last decade or so have demonstrated an important role for the RAAS in regulating cell growth and differentiation, extracellular matrix (ECM) metabolism, and inflammation in chronic diseases, such as diabetic nephropathy[22,23,24,25]. The most convincing evidence for the involvement of the RAAS in diabetic nephropathy comes from randomized, controlled clinical trials demonstrating the efficacy of angiotensin-converting enzyme (ACE) inhibitors and angiotensin II (Ang II) receptor blockers (ARBs) in slowing the progression of renal disease[26,27]. The stabilization in the number of diabetic patients going into ESRD has also largely been attributed to the efficacy of these drugs. These observations strongly support the notion that the RAAS is a major contributor in the development and progression of diabetic nephropathy.

While for decades Ang II had been regarded as the single effector molecule of the RAAS, in recent years, studies have suggested that other components of the "classical" RAAS, including aldosterone, renin, and prorenin, also have direct biological actions in the diabetic kidney[28,29,30]. In addition, recent studies have suggested that metabolites of Ang II, including Ang (1-7) and Ang (1-9), may have important biological activities in diabetic nephropathy[31,32]. Enzymes involved in their synthesis,

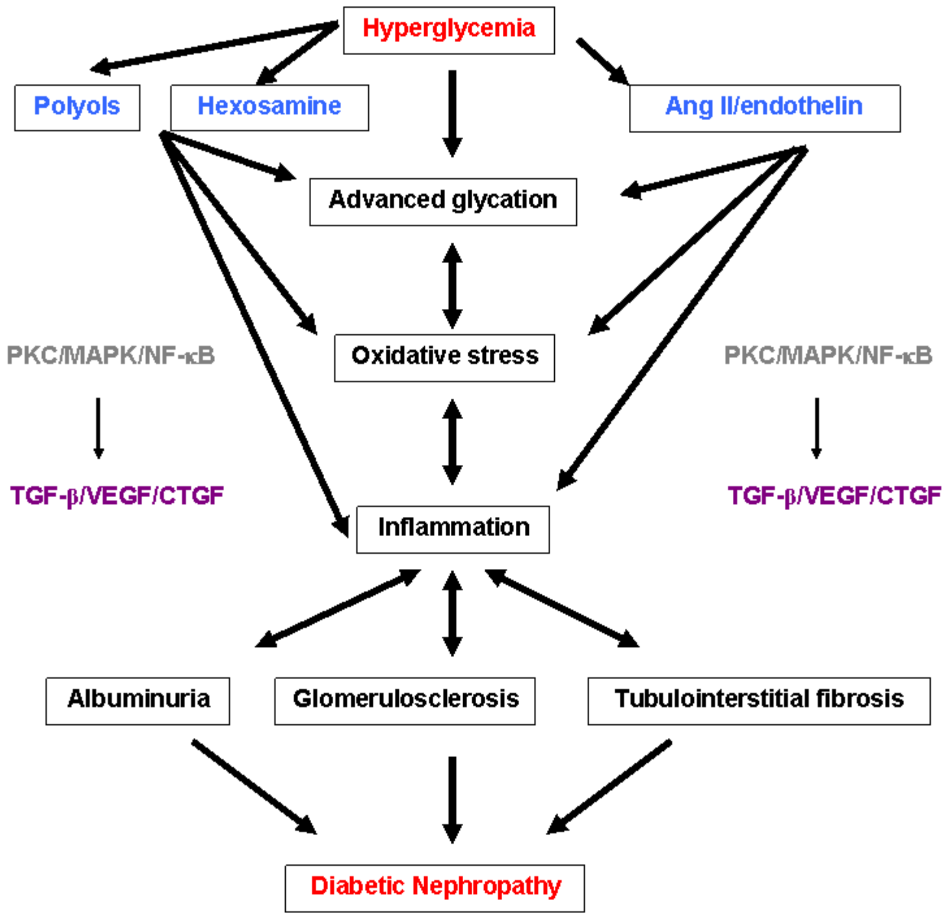

**FIGURE 1.** Interaction between metabolic and hemodynamic pathways in the pathophysiology of diabetic nephropathy.

namely ACE2 and neprilysin, have also recently been added to the increasing complexity of the RAAS[33,34]. Furthermore, several studies have suggested that alternative enzymes to ACE exist for converting Ang I into Ang II in the kidney, namely chymase[35]. Fig. 2 summarizes our current understanding of the RAAS.

## Angiotensin II (Ang II)

Ang II is one of the most potent effector molecules of the RAAS, with diverse actions in many renal cell types[36]. A large body of evidence suggests that in addition to the systemic production of Ang II, most tissue beds, including the kidney, express a local RAAS that acts independently of the circulating RAAS[36,37]. Thus, while the circulating RAAS does not seem to be activated in type 1 diabetes mellitus (T1DM) or type 2 diabetes mellitus (T2DM)[38], the renal RAAS appears to be up-regulated and contributes much to the pathophysiology of diabetic nephropathy[39]. Furthermore, most recent studies have shown that in addition to the renal RAAS, cell-specific RAAS also exists and its expression, for example, in the proximal tubules is up-regulated in the diabetic kidney[40]. These observations suggest that RAAS expression and activity are under extremely complex regulation. Moreover, these observations explain why ACE inhibitors and ARBs are effective in the treatment of diabetic nephropathy, despite the fact that circulating RAAS does not seem to be altered in the setting of diabetes.

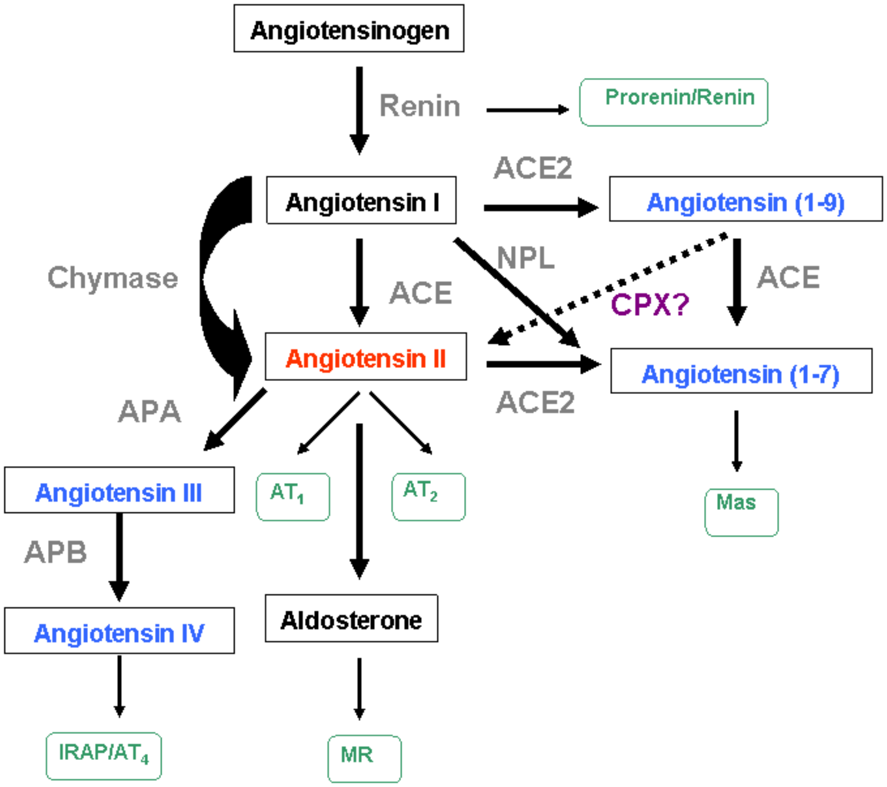

**FIGURE 2.** The current view of the RAAS showing the "classical" pathway of Ang II formation and the more recently identified enzymes, metabolites, and receptors of the RAAS. Abbreviations: ACE: angiotensin converting enzyme; ACE2: angiotensin converting enzyme-2; $AT_1$: angiotensin II type 1 receptor; $AT_2$: angiotensin II type 2 receptor; APA: aminopeptidase A; APB: aminopeptidase B; CPX: unknown carboxypeptidase; IRAP: insulin-regulated aminopeptidase; MR: mineralocorticoid receptor; NPL: neprilysin; *Mas* protooncogene; $AT_4$: angiotensin IV receptor

In the kidney, Ang II mediates its actions by signaling through its two receptor subtypes, the $AT_1$ and the $AT_2$ receptor. The $AT_1$ receptor is widely distributed throughout the kidney[37,41], while the distribution of the $AT_2$ receptor is restricted to glomerular endothelial cells and tubular epithelial cells in the cortex, interstitial, and tubular cells in the outer medulla, and inner medullary collecting duct cells[42]. It is generally believed that $AT_1$ and $AT_2$ receptors mediate the opposing effects of Ang II such that, for example, activation of $AT_1$ receptors leads to renal vasoconstriction, sodium retention, and cell proliferation[41,43,44], while activation of $AT_2$ receptors leads to vasodilatation, natriuresis, and inhibition of cell proliferation[45,46,47]. Surprisingly, the expression of renal $AT_1$ receptors has been reported to be decreased in diabetic nephropathy[48], suggesting that it may be the sensitivity of the $AT_1$ receptors to Ang II, rather than the level of its expression, that mediates the deleterious effects of Ang II activation of the $AT_1$ receptor[39,49]. Indeed, both early hyperfiltration as well as later decline in GFR associated with diabetic nephropathy is thought to be associated with $AT_1$ receptor–mediated alterations in vascular responsiveness to Ang II[7,50,51]. In addition to its hemodynamic effects, it is becoming increasingly apparent that Ang II, via the $AT_1$ receptor, contributes to the development and progression of diabetic renal structural injury. In cultured mesangial, proximal tubular, and interstitial cells, Ang II increases cell proliferation and ECM synthesis[52,53,54,55]. These actions of Ang II have mainly been attributed to stimulation of a number of profibrotic cytokines, such as TGF-β, VEGF, PDGF, and CTGF, as well as activation of downstream signaling pathways involving PKC and NF-κB[55,56,57,58], ultimately leading to glomerulosclerosis and tubulointerstitial fibrosis. Ang II, via the $AT_1$ receptor, has also been shown to be a potent proinflammatory agent, activating differentiation and proliferation of monocytes and macrophages via stimulating the expression of monocyte chemoattractant protein-1 (MCP-1), osteopontin, and RANTES[59,60,61]. In addition, Ang II has been reported to activate metabolic pathways leading to accumulation of advanced glycation end-products[62], which in turn contribute to renal injury.

The importance and contribution of $AT_2$ receptors in the diabetic kidney is less well understood and there is still much controversy regarding the levels of expression of $AT_2$ receptors in the diabetic kidney. Both decreases[42,48] and increases[45] in the level of expression of $AT_2$ receptors have been reported in experimental models of diabetic nephropathy. The decrease in $AT_2$ receptor expression supports the concept that $AT_2$ receptors may mediate the beneficial effects of Ang II and that these beneficial effects may be lost in diabetic nephropathy due to down-regulation of $AT_2$ receptor expression. The increase in $AT_2$ receptor expression in obese Zucker rats, a model of T2DM, has been reported to mediate the natriuretic affects of an $AT_1$ receptor antagonist[45]. These findings indicate that $AT_2$ receptors may compensate for the enhanced fluid intake observed in streptozotocin (STZ)-induced diabetic rats. In addition, $AT_2$ receptors have been shown to promote cell differentiation and apoptosis[63], thus offering protection against glomerulosclerosis and tubulointerstitial fibrosis associated with diabetic nephropathy. Further supporting the importance for $AT_2$ receptors in diabetic nephropathy is the recent report of the polymorphism of the $AT_2$ receptor gene that is associated with the decline in renal function and premature aging of the arterial tree in patients with type I diabetes[64].

It is important to note that not all studies are in agreement regarding the levels of expression of not only $AT_1$ and $AT_2$ receptors in the diabetic kidney, but also other components of the RAAS. The most likely explanation for these disparate findings is the experimental models of diabetic nephropathy these studies have been performed in, duration of diabetes, as well as the techniques used for assessing the levels of expression of various components of the RAAS. However, studies have also suggested that rather than the changes in the level of expression, diabetes may alter the distribution of the components of the RAAS within various kidney compartments. In an experimental model of diabetic nephropathy, proximal tubule ACE activity was reported to be reduced, but ACE immunostaining intensity was enhanced in glomeruli and renal vasculature[65]. These observations once again stress the importance of not just the renal RAAS, but also coexistence of cell-specific RAASs in the pathophysiology of diabetic nephropathy. Furthermore, these cell-specific expressions may be important in determining cell-specific effects of the RAAS, such as that albuminuria and renal fibrosis associated with diabetic nephropathy, for example, may be regulated by Ang II through different signaling pathways in different cell types.

Despite the above-mentioned controversies regarding levels of expression of the RAAS in diabetic nephropathy, several ACE inhibitors and ARBs have been shown to slow the progression of diabetic nephropathy. ACE inhibitors such as enalapril and lisinopril have clearly been shown to offer renoprotection in both experimental models of diabetic nephropathy as well as in humans[66,67,68]. The beneficial effects of ACE inhibitors may not only be related to their ability to inhibit Ang II formation, but also increase Ang (1-7) formation, which on its own may be beneficial (see discussion below). However, ACE inhibition may not be sufficient to block Ang II formation completely, as Ang II may be formed via ACE-independent mechanisms involving chymase, the expression of which is increased in the diabetic kidney[69]. In addition, ACE inhibition may also be associated with increased renin levels, which may be detrimental to the diabetic kidney. Furthermore, long-term use of ACE inhibitors is associated with frequent side effects, such as cough and angioedema[70]. Thus, while inhibiting ACE activity is beneficial to a degree, additional levels of inhibiting the RAAS, such as ARB, are needed to protect the kidney in the setting of diabetes.

Increasing evidence suggests that alternative pathways to ACE exist for Ang II generation in target tissues, including the kidney[71,72]. The most prominent of these alternative pathways is thought to be chymase dependent, as Ang II formation is substantially blocked by chymase inhibitors, such as chymostatin, NK3201, and CD41[73,74]. Recent studies have reported that chymase is markedly up-regulated in mesangial cells, mast cells, and vascular smooth muscle cells in experimental models of diabetic nephropathy[75,76]. These observations indicate that the chymase-dependent, Ang II–generating system may play an important role in the pathophysiology of diabetic nephropathy. Future studies are needed to determine the potential renoprotective effects of chymase inhibitors alone or in combination with other therapies blocking the RAAS in the diabetic kidney in humans.

The Reduction of Endpoints in Non-insulin dependent diabetes mellitus with the Angiotensin Antagonist Losartan (RENAAL)[26] and the Irbesartan Diabetic Nephropathy Trial (IDNT)[26,27] have both reported renoprotective effects of inhibiting the $AT_1$ receptor in the diabetic kidney. The Olmesartan Reducing Incidence of ESRD in diabetic Nephropathy Trial (ORIENT) is currently ongoing and is also expected to show similar renoprotective effects[77]. The beneficial effects of ARBs may not only lie in the fact that they block the $AT_1$ receptor, but that in the process, they unmask the protective effects of the $AT_2$ receptor.. On the down side, ARBs do not inhibit Ang II formation, which may potentially be able to exert its detrimental effects through an as-yet-unknown receptor. Thus, the combination therapy of ACE inhibitors and ARBs would be the path of a more complete blockade of the RAAS and thus more effective in attenuating diabetic nephropathy. Indeed, both clinical trials and experimental studies have reported the superiority of the combination therapy over either ACE inhibitors or ARBs alone in diabetic nephropathy[78,79,80,81]. However, despite the success of the combination therapy in slowing the progression of diabetic nephropathy, there is still room for improvement of therapeutic strategies relating to the RAAS. Given the existence of the renal as well as cell-specific RAAS, it is conceivable that the ultimate beneficial effects of inhibiting the RAAS in the diabetic kidney would come from inhibition at both the tissue and level of a single cell. However, developing tissue and cell-specific pharmacological inhibitors of the RAAS may, at least at this stage, be far from realistically possible.

## Renin and Prorenin

Part of the reason why ACE inhibition is only partially effective in attenuating the progression of diabetic nephropathy may be due to tissue renin accumulation, which, in turn, may lead to higher renal Ang II formation via renin-dependent, but ACE-independent, pathways[82]. Interestingly though, plasma renin levels and activity are low in patients with T2DM, while renal levels are high in the diabetic kidney, especially in the damaged tubules[40,83]. This increase in proximal tubule renin has been suggested to be responsible for the increased local formation of Ang II, which subsequently leads to tubular injury and tubulointerstitial fibrosis. Thus, renin inhibition has been identified as an independent therapeutic target, not only in the treatment of diabetic nephropathy, but chronic renal disease in general. Aliskiren is the first nonpeptide, orally active, renin inhibitor approved by the FDA[70]. Thus far, aliskiren monotherapy has been shown to provide antihypertensive efficacy and placebo-like tolerability in patients with hypertension[30,70]. While no data on the efficacy of aliskiren in the treatment of diabetic nephropathy in humans are available as yet, a recent experimental study has shown renoprotective effects of aliskiren through reducing albuminuria and renal structural injury[84].

Prorenin is a precursor of renin that, under normal physiological conditions, lacks renin enzymatic activity, i.e., it cannot generate Ang I from angiotensinogen[85]. Plasma prorenin concentrations have been shown to be elevated in T2DM, in particular in those patients with microalbuminuria[86], suggesting that prorenin may contribute to the progression of diabetic nephropathy. Both prorenin and renin have recently been identified as having direct actions on target cells in the kidney via binding to the membrane-associated protein, now called prorenin/renin receptor[87,88]. The prorenin/renin receptor is predominantly localized to mesangial cells, distal tubules, glomeruli, and the macula densa[88,89]. Binding of prorenin/renin to its receptor has been shown to have profibrotic effects via activation of the TGF-β pathway, even in the presence of an ARB (Ref 23 of Huang et al). These findings suggest that renin and prorenin may have direct cellular effects independent of Ang II formation. A site-specific binding protein (called “decoy peptide”) has recently been found to prevent prorenin activation on binding to the prorenin/renin receptor[88]. In an experimental model of diabetic nephropathy, the “decoy peptide” has been shown to attenuate glomerulosclerosis and tubulointerstitial fibrosis, both directly[90,91] and indirectly[91], via inhibition of local Ang II formation. These observations support the concept of the importance for renin and prorenin in the pathophysiology of diabetic nephropathy in concert with and independent of Ang II. Further studies are needed to evaluate whether renin inhibition and blockade of the prorenin/renin receptor, in addition to ACE inhibition and/or ARB, may provide synergistic renoprotective effects in diabetic nephropathy in humans.

## Aldosterone

The concept of "aldosterone escape" in the context of ACE inhibition is well recognized. Long-term ACE inhibition or ARBs are associated with increases in plasma aldosterone levels in some patients after an initial reduction or unchanged levels[28,92]. These observations led to the belief that one of the underlying reasons for the incomplete renoprotection offered by ACE inhibitors or ARBs is elevation in aldosterone levels. In addition, in the past decade, a broader perspective has also emerged regarding aldosterone's actions in the kidney. Aldosterone is now not just believed to be involved in water and electrolyte balance, but that it has direct actions on target cells leading to renal injury. These findings suggest that aldosterone inhibition may be an effective treatment for diabetic nephropathy. Indeed, in experimental models, spironolactone, a mineralocorticoid receptor (MR) blocker, reduces albuminuria, glomerulosclerosis, macrophage infiltration, renal MCP-1 synthesis, and expression of its upstream transcription factor NF-κB[93]. In patients with T2DM complicated by diabetic nephropathy, spironolactone decreases albuminuria and plasminogen PAI-1[94]. Addition of spironolactone to an ACE inhibitor or ARB also reduced proteinuria, which, in part, relates to the more pronounced reduction in GFR[28,95,96]. These studies concluded that the combination therapy of aldosterone blockade with ACE inhibition or ARB confers additional renoprotection in patients with diabetic nephropathy. While both experimental and clinical observations indicate that aldosterone blockade may be a potential therapeutic target in diabetic nephropathy, it should be stressed that long-term administration of MR blockers is associated with a major adverse effect of hyperkalemia[97].

## Metabolites of Ang II: Ang (1-7), Ang (1-9), and Ang IV

Studies in the last several years have shown that Ang I can be metabolized to Ang (1-7) by enzymes such as neprilysin[98,99,100]. In addition, Ang (1-7) can also be formed as a result of metabolism of Ang (1-9) by ACE and metabolism of Ang II by ACE2[98,99,100]. Ang (1-7), via binding to its Mas receptor[101], is a potent vasodilator that has been shown to have antihypertensive, anti-inflammatory, and antiproliferative properties in several tissues, including the kidney[99,102,103]. These actions of Ang (1-7), in essence, oppose the actions of $AT_1$ receptor–mediated effects of Ang II. To date, however, very few studies have examined the direct effects of Ang (1-7) in the diabetic kidney. In the STZ-induced Wistar rat kidneys, there is a down-regulation in the expression of Ang (1-7) mRNA compared with the nondiabetic rat, but differences in renal Ang (1-7) levels were not observed[104]. These observations suggest that diabetes may be associated with a down-regulation of renal Ang (1-7) levels. In the same experimental models, treatment with Ang (1-7) and/or Ang (1–7) receptor Mas agonist AVE-0991 reduces albuminuria and prevents diabetes-induced, abnormal vascular responsiveness to norepinephrine, endothelin-1, and Ang II[31]. These observations support the concept for Ang (1-7) as a renoprotective agent in diabetes. Future studies are needed to address whether similar renoprotective effects of Ang (1-7) and Mas receptor analogues extend to human diabetic nephropathy.

Perhaps the most interesting aspect or, at least, the element of the Ang II/Ang (1-7) axis that has received the most attention in the recent years is ACE2. Since ACE2 degrades Ang II to Ang (1-7), it is now thought that ACE2 acts as a functional clearance mechanism for Ang II, acting as a counter-regulatory manner to ACE by shifting the balance between Ang II and Ang (1-7)[100]. In the healthy kidney, ACE2 is highly expressed in proximal tubules, endothelial and smooth muscle cells of renal vessels, and podocytes[105,106]. Experimental studies evaluating the expression of ACE2 in the diabetic kidney have thus far reported conflicting findings. In the STZ-induced diabetic rat, ACE2 expression has been shown to be decreased in the proximal tubules, while glomerular expression was increased after 24 weeks of diabetes[34]. In the *db/db* mouse, a model of diabetic nephropathy associated with T2DM, both ACE2 mRNA and protein expression have been shown to be up-regulated in the renal cortex in early diabetes[105,107]. Thus, it appears that in early stages of diabetic kidney disease, ACE2 is up-regulated, probably as a protective mechanism against the increase in ACE-dependent Ang II formation and subsequent development of diabetic nephropathy. Indeed, increases in ACE2 expression are coupled with decreases in ACE expression[105,107]. As a result of prolonged hyperglycemia and consequent activation of proinflammatory and profibrotic pathways, ACE2 expression becomes down-regulated and may contribute to the disease progression. In support of the concept that down-regulation of ACE2 may contribute to the disease is a study showing that *db/db* mice infused with an ACE2 antagonist developed albuminuria and glomerulosclerosis[105]. Interestingly, polymorphisms in the ACE2 gene have been reported not to be associated with the decline in renal function and progression of proteinuria in diabetic nephropathy associated with T1DM[108]. Similar studies in patients with diabetic nephropathy associated with T2DM have thus far not been performed.

The beneficial effects of ACE inhibitors and ARBs have been attributed, in part, to increasing renal ACE2 activity[109]. At the same time, since ACE inhibition can also reduce Ang (1-7) formation, this may partially abolish the renoprotective effect of ACE inhibitors and/or ARBs. While further studies are needed to examine, in more detail, the role of ACE2 in the pathophysiology of diabetic nephropathy, based on the currently available information, it is conceivable that ACE2 agonists alone or combined with ACE inhibitors and/or ARBs may be useful in the treatment of diabetic nephropathy.

Ang I can be degraded into Ang (1-9) by ACE2[98]. While the role for Ang (1-9) in diabetic nephropathy has thus far not been properly evaluated, it may be expected that generation of Ang (1-9) from Ang I may be beneficial, as Ang (1-9) may be converted into a vasodilator Ang (1-7). Interestingly, increased conversion of exogenous Ang (1-9) into Ang II in glomerular extracts from diabetic rats has been reported, with the reaction being catalyzed by an as-yet-unknown carboxypeptidase (CPX)[32]. These observations suggest that increased levels of Ang (1-9) may, in fact, be detrimental, as they may provide an additional pathway for Ang II formation in the diabetic kidney. Conversion of Ang I to Ang (1-9) is generally thought not to be of significance under normal physiological conditions, but rather under conditions that raise Ang II levels, e.g., ARB treatment[110]. Further studies are needed to evaluate the significance of Ang (1-9) in the diabetic kidney properly, although based on the current knowledge, it appears that attenuation of the Ang (1-9)-dependent pathway of Ang II formation may be a potential novel therapeutic target in the treatment of diabetic nephropathy.

Recent studies have identified the importance for N-terminal angiotensin degradation product, Ang IV, in cardiovascular disease[111]. Ang IV is generated by degradation of Ang II by aminopeptidase N (APN) in response to tissue injury, suggesting that elevated levels of Ang IV can be found under pathophysiological conditions. Ang IV then binds to the $AT_4$ receptor, which has recently been identified as an insulin-regulated aminopeptidase (IRAP). Ang IV has been shown to regulate cell growth in cardiac fibroblasts, endothelial cells, and vascular smooth muscle cells. In the kidney, Ang IV has been shown to have both vasoconstrictor and vasodilatory effects, and induces PAI-1 expression in an experimental model of hypertension[112]. However, to date, no studies have examined the role of $AT_4$/IRAP in the diabetic kidney. Given its importance in regulating renal function in hypertension, it is likely that future studies will unravel the role for Ang IV/IRAP in diabetic nephropathy.

## Endothelin

Endothelins are potent vasoconstrictors with proinflammatory, mitogenic, and profibrotic properties in the kidney[113,114,115,116]. The endothelin family consists of three distinct isoforms, namely endothelin-1, endothelin-2, and endothelin-3, with endothelin-1 being the most potent in terms of its renal actions. Endothelin-1 exerts its renal actions mainly through its two receptor subtypes: $ET_A$ and $ET_B$[114,117,118]. In the kidney, both of these receptor subtypes are widely distributed[118,119], suggesting that endothelin-1 plays a role in modulating a number of renal functions under normal physiological conditions. More interestingly, increasing evidence suggests that endothelin-1 is also an important mediator of renal function under pathophysiological conditions, including diabetic nephropathy. Selective $ET_A$ receptor antagonists as well as combined $ET_A/ET_B$ receptor antagonists prevent the development of hypertension, renal vasoconstriction, inflammation, proteinuria, glomerulosclerosis, and tubulointerstitial fibrosis in experimental models of diabetic nephropathy[12,83,120,121,122]. Inflammation and fibrosis appear to be mediated via both $ET_A$ and $ET_B$ receptors, while vasoconstrictor actions of endothelin-1 are primarily mediated via $ET_A$ receptors[117]. $ET_B$ receptors have also been suggested to be involved in endothelin-1 clearance. This concept is supported by a recent report showing that diabetic mice lacking the $ET_B$ receptor develop low-renin hypertension and progressive renal failure, and have increased endothelin-1 levels[123]. In other experimental models, enhanced renal synthesis of endothelin-1 has been observed in diabetes[120,123,124], while high glucose conditions *in vitro*, as well as Ang II, increase endothelin-1 mRNA and protein levels[125,126]. Experimental models of diabetic nephropathy have also shown increases in $ET_A$ receptor expression[127,128]. *In vitro* studies further support the role for endothelin-1 under high glucose conditions. In cultured proximal tubule cells, endothelin-1 increases ECM protein synthesis by activating MAPK and PKC, and increasing production of reactive oxygen species[125,129,130,131]. These findings support the concept that inhibition of endothelin may offer renoprotection in the setting of diabetes. Further clinical evaluation of endothelin-1 receptor antagonism either alone or in combination with inhibiting the RAAS in diabetic nephropathy is warranted.

## Urotensin II

Urotensin II is a powerful vasoconstrictor with potency greater than that of endothelin-1; however, its vasoconstrictive properties are restricted to certain regional vascular beds[132]. Not surprisingly, given its vasoconstrictive actions, urotensin II has been shown to be a potent growth factor in porcine renal epithelial cells[133], vascular smooth muscle cells[134,135], and myocardial cells[136]. Recent experimental and clinical studies have revealed increased expression of urotensin II and urotensin II receptors in diabetic nephropathy[11], suggesting a potential role for urotensin II in diabetic renal diseases. A series of peptide and nonpeptide urotensin II receptor ligands have been shown to be effective in antagonizing the effects of urotensin II in the cardiovascular system; however, their effect in the diabetic kidney have thus far not been evaluated. While further studies are needed to understand the actions of urotensin II in the diabetic kidney fully, based on the current knowledge, it is likely that the interaction between urotensin II and other endogenous vasoactive hormones may play a significant role in the pathophysiology of diabetic nephropathy.

## Kallikrein-Kinin System (KKS)

Similar to the RAAS, the KKS is a complex multienzyme system that has a circulating as well as a tissue/renal component[137,138]. The KKS consists of a precursor kininogen, which is cleaved by kallikrein enzymes, leading to the formation of the biologically active kinins[139]. One of the most potent of kinins is bradykinin, which mediates its renal actions via signaling through bradykinin B1 and B2 receptor subtypes[140]. Kinins are endogenous vasodilators that have been shown to play an important role in the regulation of renal function under both normal physiological as well as pathophysiological conditions, such as diabetic nephropathy. Specifically, kinins antagonize Ang II–induced vasoconstriction and sodium retention via increasing cP450 metabolites and nitric oxide[141]. In addition, bradykinin, via the B2 receptor, has been shown to have antiproliferative and anti-inflammatory effects in the kidney[142,143]. In support of the beneficial effects of B2 receptors in the diabetic kidney is the study showing that mice lacking the bradykinin B2 receptor are more susceptible to the development of diabetic nephropathy[144]. Interestingly, a very recent study showed the opposite effects of targeted deletion of B2 receptors; these mice were protected from the development of diabetic nephropathy[145]. The most likely explanation from these disparate findings is the difference in mouse strains and duration of diabetes. It is conceivable that the absence of B2 receptors in early diabetes may have beneficial effects in terms of attenuating hyperfiltration and glomerular hypertrophy. However, prolonged absence of B2 receptors may be detrimental, setting a stage for the development of hypertension, inflammation, and renal injury. In support of this concept is the report on increased levels of bradykinin in early diabetes in the STZ-induced rat kidney, and decreased bradykinin levels in more advanced stages of the disease[146]. Future studies are needed to determine the efficacy of selective nonpeptide B2 receptor agonists in the treatment of diabetic nephropathy.

Kinins are rapidly inactivated by several peptidases, including ACE and neutral endopeptidase (NEP)[147]. The efficacy of ACE inhibitors in attenuating the progression of diabetic nephropathy has been attributed, in part, to its ability to inhibit kinin degradation. Thus, there has been a great deal of interest in developing more specific inhibitors of kininases to extend the potency of kinins in mediating vasodilatation and antiproliferation in a disease setting. A dual ACE/NEP inhibitor, omapatrilat, has been shown to prevent albuminuria, glomerulosclerosis, tubulointerstitial fibrosis, and hypertension in experimental models of diabetic nephropathy[148,149,150]. These studies concluded that the dual inhibition of ACE and NEP may offer more superior renoprotection to the diabetic kidney than therapy with an ACE inhibitor. In particular, omapatrilat has been shown to be more effective in reducing blood pressure than an ACE inhibitor[149]. Furthermore, the increased renoprotective effect of omapatrilat may also be due, in part, to increased levels of bradykinin-stimulated B2 receptors. Future studies are needed to evaluate whether a similar synergistic renoprotection can be achieved in the treatment of diabetic nephropathy in humans.

## CONCLUSION

Until the recent past, the treatment of diabetic nephropathy has relied on tight control of hyperglycemia, hypertension, and the inhibition of the RAAS. While these treatments have proven efficacious and have been credited for the recently reported stabilization in the number of patients with diabetic nephropathy progressing to ESRD, additional studies are needed to evaluate the potential renoprotective effects of other vasoactive hormones. The recognition of the importance of the classical components of the RAAS in addition to the more novel elements of this hormonal pathway are just some of the candidates for the development of new therapeutic treatments for diabetic nephropathy. It is most likely that resolution of diabetic nephropathy will require synergistic therapies to target multiple mediators of this disease.

## References

1. de Zeeuw, D., Ramjit, D., Zhang, Z., Ribeiro, A.B., Kurokawa, K., Lash, J.P., Chan, J., Remuzzi, G., Brenner, B.M., and Shahinfar, S. (2006) Renal risk and renoprotection among ethnic groups with type 2 diabetic nephropathy: a post hoc analysis of RENAAL. *Kidney Int.* **69**, 1675–1682.
2. Remuzzi, G., Macia, M., and Ruggenenti, P. (200) Prevention and treatment of diabetic renal disease in type 2 diabetes: the BENEDICT study. *J. Am. Soc. Nephrol.* **17**, S90–S97.
3. U.S. Renal Data System (2006) USRDS 2006 Annual Data Report. NIDDK The National Institutes of Health.
4. Thomson, S.C., Vallon, V., and Blantz, R.C. (2004) Kidney function in early diabetes: the tubular hypothesis of glomerular filtration. *Am. J. Physiol. Renal Physiol.* **286**, F8–F15.
5. Hostetter, T.H. (2003) Hyperfiltration and glomerulosclerosis. *Semin. Nephrol.* **23**, 194–199.
6. Caramori, M.L. and Mauer, M. (2003) Diabetes and nephropathy. *Curr. Opin. Nephrol. Hypertens.* **12**, 273–282.
7. Leon, C.A. and Raij, L. (2005) Interaction of haemodynamic and metabolic pathways in the genesis of diabetic nephropathy. *J. Hypertens.* **23**, 1931–1937.
8. Brownlee, M. (2001) Biochemistry and molecular cell biology of diabetic complications. *Nature* **414**, 813–820.
9. King, G.L. and Brownlee, M. (1996) The cellular and molecular mechanisms of diabetic complications. *Endocrinol. Metab. Clin. North Am.* **25**, 255–270.

10. Wolf, G. and Ziyadeh, F.N. (2007) Cellular and molecular mechanisms of proteinuria in diabetic nephropathy. *Nephron Physiol.* **106**, 26–31.
11. Langham, R.G., Kelly, D.J., Gow, R.M., Zhang, Y., Dowling, J.K., Thomson, N.M., and Gilbert, R.E. (2004) Increased expression of urotensin II and urotensin II receptor in human diabetic nephropathy. *Am. J. Kidney Dis.* **44**, 826–831.
12. Cosenzi, A., Bernobich, E., Trevisan, R., Milutinovic, N., Borri, A., and Bellini, G. (2003) Nephroprotective effect of bosentan in diabetic rats. *J. Cardiovasc. Pharmacol.* **42**, 752–756.
13. Jacobsen, P. and Parving, H.H. (2004) Beneficial impact on cardiovascular risk factors by dual blockade of the renin-angiotensin system in diabetic nephropathy. *Kidney Int. Suppl.* **92**, S108–S110.
14. Cooper, M. and Boner, G. (2004) Dual blockade of the renin-angiotensin system in diabetic nephropathy. *Diabet. Med.* **21(Suppl 1),** 15–18.
15. Hanes, D.S., Nahar, A., and Weir, M.R. (2004) The tissue renin-angiotensin-aldosterone system in diabetes mellitus. *Curr. Hypertens. Rep.* **6**, 98–105.
16. Noh, H. and King, G.L. (2007) The role of protein kinase C activation in diabetic nephropathy. *Kidney Int. Suppl.* **106**, S49–S53.
17. Zhu, Y., Usui, H.K., and Sharma, K. (2007) Regulation of transforming growth factor beta in diabetic nephropathy: implications for treatment. *Semin. Nephrol.* **27**, 153–160.
18. Tomlinson, D.R. (1999) Mitogen-activated protein kinases as glucose transducers for diabetic complications. *Diabetologia* **42**, 1271–1281.
19. Iwamoto, M., Mizuiri, S., Arita, M., and Hemmi, H. (2006) Nuclear factor-kappaB activation in diabetic rat kidney: evidence for involvement of P-selectin in diabetic nephropathy. *Tohoku J. Exp. Med.* **206**, 163–171.
20. Weir, M.R. and Dzau, V.J. (1999) The renin-angiotensin-aldosterone system: a specific target for hypertension management. *Am. J. Hypertens.* **12**, S205–S213.
21. Navar, L.G., Imig, J.D., Zou, L., and Wang, C.T. (1997) Intrarenal production of angiotensin II. *Semin. Nephrol.* **17**, 412–422.
22. Yoo, T.H., Li, J.J., Kim, J.J., Jung, D.S., Kwak, S.J., Ryu, D.R., Choi, H.Y., Kim, J.S., Kim, H.J., Han, S.H., et al. (2007) Activation of the renin-angiotensin system within podocytes in diabetes. *Kidney Int.* **71**, 1019–1027.
23. Huang, Y., Noble, N.A., Zhang, J., Xu, C., Border, W.A. (2007) Renin-stimulated TGF-beta1 expression is regulated by a mitogen-activated protein kinase in mesangial cells. Kidney Int. 72, 45-52.
24. Nicholas, S.B., Mauer, M., Basgen, J.M., Aguiniga, E., and Chon, Y. (2004) Effect of angiotensin II on glomerular structure in streptozotocin-induced diabetic rats. *Am. J. Nephrol.* **24**, 549–556.
25. Tone, A., Shikata, K., Ogawa, D., Sasaki, S., Nagase, R., Sasaki, M., Yozai, K., Usui, H.K., Okada, S., Wada, J. et al. (2007) Changes of gene expression profiles in macrophages stimulated by angiotensin II--angiotensin II induces MCP-2 through AT1-receptor. *J. Renin Angiotensin Aldosterone Syst.* **8**, 45–50.
26. Brenner, B.M., Cooper, M.E., de Zeeuw, D., Keane, W.F., Mitch, W.E., Parving, H.H., Remuzzi, G., Snapinn, S.M., Zhang, Z., and Shahinfar, S. (2001) Effects of losartan on renal and cardiovascular outcomes in patients with type 2 diabetes and nephropathy. *N. Engl. J. Med.* **345**, 861–869.
27. Lewis, E.J., Hunsicker, L.G., Clarke, W.R., Berl, T., Pohl, M.A., Lewis, J.B., Ritz, E., Atkins, R.C., Rohde, R., and Raz, I. (2001) Renoprotective effect of the angiotensin-receptor antagonist irbesartan in patients with nephropathy due to type 2 diabetes. *N. Engl. J. Med.* **345**, 851–860.
28. Schjoedt, K.J., Rossing, K., Juhl, T.R., Boomsma, F., Tarnow, L., Rossing, P., and Parving, H.H. (2006) Beneficial impact of spironolactone on nephrotic range albuminuria in diabetic nephropathy. *Kidney Int.* **70**, 536–542.
29. Schmieder, R.E. (2007) The potential role of prorenin in diabetic nephropathy. *J. Hypertens.* **25**, 1323–1326.
30. Sepehrdad, R., Frishman, W.H., Stier, C.T., Jr., and Sica, D.A. (2007) Direct inhibition of renin as a cardiovascular pharmacotherapy: focus on aliskiren. *Cardiol. Rev.* **15**, 242–256.
31. Benter, I.F., Yousif, M.H., Anim, J.T., Cojocel, C., and Diz, D.I. (2006) Angiotensin-(1-7) prevents development of severe hypertension and end-organ damage in spontaneously hypertensive rats treated with L-NAME. *Am. J. Physiol. Heart Circ. Physiol.* **290**, H684–H691.
32. Singh, R., Singh, A.K., and Leehey, D.J. (2005) A novel mechanism for angiotensin II formation in streptozotocin-diabetic rat glomeruli. *Am. J. Physiol. Renal Physiol.* **288**, F1183–F1190.
33. Ingelfinger, J.R. (2006) ACE2: a new target for prevention of diabetic nephropathy? *J. Am. Soc. Nephrol.* **17**, 2957–2959.
34. Tikellis, C., Johnston, C.I., Forbes, J.M., Burns, W.C., Burrell, L.M., Risvanis, J., and Cooper, M.E. (2003) Characterization of renal angiotensin-converting enzyme 2 in diabetic nephropathy. *Hypertension* **41**, 392–397.
35. Hollenberg, N.K., Fisher, N.D., and Price, D.A. (1998) Pathways for angiotensin II generation in intact human tissue: evidence from comparative pharmacological interruption of the renin system. *Hypertension* **32**, 387–392.
36. Paul, M., Poyan Mehr, A., and Kreutz, R. (2006) Physiology of local renin-angiotensin systems. *Physiol. Rev.* **86**, 747–803.
37. Carey, R.M. and Siragy, H.M. (2003) The intrarenal renin-angiotensin system and diabetic nephropathy. *Trends Endocrinol. Metab.* **14**, 274–281.
38. Wolf, G. and Ziyadeh, F.N. (1997) The role of angiotensin II in diabetic nephropathy: emphasis on nonhemodynamic mechanisms. *Am. J. Kidney Dis.* **29**, 153–163.

39. Kennefick, T.M., Oyama, T.T., Thompson, M.M., Vora, J.P., and Anderson, S. (1996) Enhanced renal sensitivity to angiotensin actions in diabetes mellitus in the rat. *Am. J. Physiol.* **271**, F595–F602.
40. Zimpelmann, J., Kumar, D., Levine, D.Z., Wehbi, G., Imig, J.D., Navar, L.G., and Burns, K.D. (2000) Early diabetes mellitus stimulates proximal tubule renin mRNA expression in the rat. *Kidney Int.* **58**, 2320–2330.
41. Zhuo, J.L. and Li, X.C. (2007) Novel roles of intracrine angiotensin II and signalling mechanisms in kidney cells. *J. Renin Angiotensin Aldosterone Syst.* **8**, 23–33.
42. Wehbi, G.J., Zimpelmann, J., Carey, R.M., Levine, D.Z., and Burns, K.D. (2001) Early streptozotocin-diabetes mellitus downregulates rat kidney AT2 receptors. *Am. J. Physiol. Renal Physiol.* **280**, F254–F265.
43. Griffin, K.A. and Bidani, A.K. (2006) Progression of renal disease: renoprotective specificity of renin-angiotensin system blockade. *Clin. J. Am. Soc. Nephrol.* **1**, 1054–1065.
44. Crowley, S.D., Gurley, S.B., and Coffman, T.M. (2007) AT(1) receptors and control of blood pressure: the kidney and more. *Trends Cardiovasc. Med.* **17**, 30–34.
45. Hakam, A.C., Siddiqui, A.H., and Hussain, T. (2006) Renal angiotensin II AT2 receptors promote natriuresis in streptozotocin-induced diabetic rats. *Am. J. Physiol. Renal Physiol.* **290**, F503–F508.
46. Duke, L.M., Evans, R.G., and Widdop, R.E. (2005) AT2 receptors contribute to acute blood pressure-lowering and vasodilator effects of AT1 receptor antagonism in conscious normotensive but not hypertensive rats. *Am. J. Physiol. Heart Circ. Physiol.* **288**, H2289–H2297.
47. Goto, M., Mukoyama, M., Suga, S., Matsumoto, T., Nakagawa, M., Ishibashi, R., Kasahara, M., Sugawara, A., Tanaka, I., and Nakao, K. (1997) Growth-dependent induction of angiotensin II type 2 receptor in rat mesangial cells. *Hypertension* **30**, 358–362.
48. Bonnet, F., Candido, R., Carey, R.M., Casley, D., Russo, L.M., Osicka, T.M., Cooper, M.E., and Cao, Z. (2002) Renal expression of angiotensin receptors in long-term diabetes and the effects of angiotensin type 1 receptor blockade. *J. Hypertens.* **20**, 1615–1624.
49. Burns, K.D. (2000) Angiotensin II and its receptors in the diabetic kidney. *Am. J. Kidney Dis.* **36**, 449–467.
50. Gurley, S.B. and Coffman, T.M. (2007) The renin-angiotensin system and diabetic nephropathy. *Semin. Nephrol.* **27**, 144–152.
51. Carmines, P.K. and Ohishi, K. (1999) Renal arteriolar contractile responses to angiotensin II in rats with poorly controlled diabetes mellitus. *Clin. Exp. Pharmacol. Physiol.* **26**, 877–882.
52. Choi, K.H., Kang, S.W., Lee, H.Y., and Han, D.S. (1996) The effects of high glucose concentration on angiotensin II- or transforming growth factor-beta-induced DNA synthesis, hypertrophy and collagen synthesis in cultured rat mesangial cells. *Yonsei Med. J.* **37**, 302–311.
53. Hsieh, T.J., Fustier, P., Zhang, S.L., Filep, J.G., Tang, S.S., Ingelfinger, J.R., Fantus, I.G., Hamet, P., and Chan, J.S. (2003) High glucose stimulates angiotensinogen gene expression and cell hypertrophy via activation of the hexosamine biosynthesis pathway in rat kidney proximal tubular cells. *Endocrinology* **144**, 4338–4349.
54. Maric, C., Zheng, W., and Walther, T. (2006) Interactions between angiotensin ll and atrial natriuretic peptide in renomedullary interstitial cells: the role of neutral endopeptidase. *Nephron Physiol.* **103**, 149–156.
55. Qi, W., Chen, X., Zhang, Y., Holian, J., Mreich, E., Gilbert, R.E., Kelly, D.J., and Pollock, C.A. (2007) High glucose induces macrophage inflammatory protein-3α in renal proximal tubule cells via a transforming growth factor-β1 dependent mechanism. *Nephrol. Dial. Transplant.* **22(11),** 3147–3153.
56. Ruiz-Ortega, M., Lorenzo, O., Ruperez, M., Konig, S., Wittig, B., and Egido, J. (2000) Angiotensin II activates nuclear transcription factor kappaB through AT(1) and AT(2) in vascular smooth muscle cells: molecular mechanisms. *Circ. Res.* **86**, 1266–1272.
57. Flyvbjerg, A. (2000) Putative pathophysiological role of growth factors and cytokines in experimental diabetic kidney disease. *Diabetologia* **43**, 1205–1223.
58. Forbes, J.M., Fukami, K., and Cooper, M.E. (2007) Diabetic nephropathy: where hemodynamics meets metabolism. *Exp. Clin. Endocrinol. Diabetes* **115**, 69–84.
59. Ruiz-Ortega, M., Bustos, C., Hernandez-Presa, M.A., Lorenzo, O., Plaza, J.J., and Egido, J. (1998) Angiotensin II participates in mononuclear cell recruitment in experimental immune complex nephritis through nuclear factor-kappa B activation and monocyte chemoattractant protein-1 synthesis. *J. Immunol.* **161**, 430–439.
60. Ricardo, S.D., Franzoni, D.F., Roesener, C.D., Crisman, J.M., and Diamond, J.R. (2000) Angiotensinogen and AT(1) antisense inhibition of osteopontin translation in rat proximal tubular cells. *Am. J. Physiol. Renal Physiol.* **278**, F708–F716.
61. Wolf, G., Ziyadeh, F.N., Thaiss, F., Tomaszewski, J., Caron, R.J., Wenzel, U., Zahner, G., Helmchen, U., and Stahl, R.A. (1997) Angiotensin II stimulates expression of the chemokine RANTES in rat glomerular endothelial cells. Role of the angiotensin type 2 receptor. *J. Clin. Invest.* **100**, 1047–1058.
62. Forbes, J.M., Cooper, M.E., Oldfield, M.D., and Thomas, M.C. (2003) Role of advanced glycation end products in diabetic nephropathy. *J. Am. Soc. Nephrol.* **14**, S254–S258.
63. Kumar, D., Zimpelmann, J., Robertson, S., and Burns, K.D. (2004) Tubular and interstitial cell apoptosis in the streptozotocin-diabetic rat kidney. *Nephron Exp. Nephrol.* **96**, 77–88.
64. Pettersson-Fernholm, K., Frojdo, S., Fagerudd, J., Thomas, M.C., Forsblom, C., Wessman, M., and Groop, P.H. (2006) The AT2 gene may have a gender-specific effect on kidney function and pulse pressure in type I diabetic patients. *Kidney Int.* **69**, 1880–1884.

65. Anderson, S., Jung, F.F., and Ingelfinger, J.R. (1993) Renal renin-angiotensin system in diabetes: functional, immunohistochemical, and molecular biological correlations. *Am. J. Physiol.* **265**, F477–F486.
66. Rossing, K., Jacobsen, P., Pietraszek, L., and Parving, H.H. (2003) Renoprotective effects of adding angiotensin II receptor blocker to maximal recommended doses of ACE inhibitor in diabetic nephropathy: a randomized double-blind crossover trial. *Diabetes Care* **26**, 2268–2274.
67. Kennefick, T.M. and Anderson, S. (1997) Role of angiotensin II in diabetic nephropathy. *Semin. Nephrol.* **17**, 441–447.
68. Parving, H.H. (1996) Effects of ACE inhibitors on renal function in incipient and overt diabetic nephropathy. *J. Diabetes Complications* **10**, 133–135.
69. Huang, X., Chen, W., Truong, L., and Lan, H. (2003) Chymase is upregulated in diabetic nephropathy: implications for an alternative pathway of angiotensin II-mediated diabetic renal and vascular disease. *J. Am. Soc. Nephrol.* **14**, 1738–1747.
70. Aulakh, G.K., Sodhi, R.K., and Singh, M. (2007) An update on non-peptide angiotensin receptor antagonists and related RAAS modulators. *Life Sci.* **81**, 615–639.
71. Dell'Italia, L.J. and Husain, A. (2002) Dissecting the role of chymase in angiotensin II formation and heart and blood vessel diseases. *Curr. Opin. Cardiol.* **17**, 374–379.
72. Miyazaki, M. and Takai, S. (2001) Local angiotensin II-generating system in vascular tissues: the roles of chymase. *Hypertens. Res.* **24**, 189–193.
73. Richard, V., Hurel-Merle, S., Scalbert, E., Ferry, G., Lallemand, F., Bessou, J.P., and Thuillez, C. (2001) Functional evidence for a role of vascular chymase in the production of angiotensin II in isolated human arteries. *Circulation* **104**, 750–752.
74. Takai, S., Yuda, A., Jin, D., Nishimoto, M., Sakagichi, M., Sasaki, S., and Miyazaki, M. (2000) Inhibition of chymase reduces vascular proliferation in dog grafted veins. *FEBS Lett.* **467**, 141–144.
75. Huang, X.R., Chen, W.Y., Truong, L.D., and Lan, H.Y. (2003) Chymase is upregulated in diabetic nephropathy: implications for an alternative pathway of angiotensin II-mediated diabetic renal and vascular disease. *J. Am. Soc. Nephrol.* **14**, 1738–1747.
76. Jones, S.E., Gilbert, R.E., and Kelly, D.J. (2004) Tranilast reduces mesenteric vascular collagen deposition and chymase-positive mast cells in experimental diabetes. *J. Diabetes Complications* **18**, 309–315.
77. Imai, E., Ito, S., Haneda, M., Chan, J.C., and Makino, H. (2006) Olmesartan reducing incidence of endstage renal disease in diabetic nephropathy trial (ORIENT): rationale and study design. *Hypertens. Res.* **29**, 703–709.
78. Mogensen, C.E., Neldam, S., Tikkanen, I., Oren, S., Viskoper, R., Watts, R.W., and Cooper, M.E. (2000) Randomised controlled trial of dual blockade of renin-angiotensin system in patients with hypertension, microalbuminuria, and non-insulin dependent diabetes: the candesartan and lisinopril microalbuminuria (CALM) study. *BMJ* **321**, 1440–1444.
79. Cao, Z., Bonnet, F., Davis, B., Allen, T.J., and Cooper, M.E. (2001) Additive hypotensive and anti-albuminuric effects of angiotensin-converting enzyme inhibition and angiotensin receptor antagonism in diabetic spontaneously hypertensive rats. *Clin. Sci. (Lond.)* **100**, 591–599.
80. Wilkinson-Berka, J.L., Gibbs, N.J., Cooper, M.E., Skinner, S.L., and Kelly, D.J. (2001) Renoprotective and anti-hypertensive effects of combined valsartan and perindopril in progressive diabetic nephropathy in the transgenic (mRen-2)27 rat. *Nephrol. Dial. Transplant.* **16**, 1343–1349.
81. Jacobsen, P., Andersen, S., Rossing, K., Jensen, B.R., and Parving, H.H. (2003) Dual blockade of the renin-angiotensin system versus maximal recommended dose of ACE inhibition in diabetic nephropathy. *Kidney Int.* **63**, 1874–1880.
82. Athyros, V.G., Mikhailidis, D.P., Kakafika, A.I., Tziomalos, K., and Karagiannis, A. (2007) Angiotensin II reactivation and aldosterone escape phenomena in renin-angiotensin-aldosterone system blockade: is oral renin inhibition the solution? *Expert. Opin. Pharmacother.* **8**, 529–535.
83. Kelly, D.J., Skinner, S.L., Gilbert, R.E., Cox, A.J., Cooper, M.E., and Wilkinson-Berka, J.L. (2000) Effects of endothelin or angiotensin II receptor blockade on diabetes in the transgenic (mRen-2)27 rat. *Kidney Int.* **57**, 1882–1894.
84. Kelly, D.J., Zhang, Y., Moe, G., Naik, G., and Gilbert, R.E. (2007) Aliskiren, a novel renin inhibitor, is renoprotective in a model of advanced diabetic nephropathy in rats. *Diabetologia* **50**, 2398–2404.
85. Oliver, J.A. (2006) Receptor-mediated actions of renin and prorenin. *Kidney Int.* **69**, 13–15.
86. Daneman, D., Crompton, C.H., Balfe, J.W., Sochett, E.B., Chatzilias, A., Cotter, B.R., and Osmond, D.H. (1994) Plasma prorenin as an early marker of nephropathy in diabetic (IDDM) adolescents. *Kidney Int.* **46**, 1154–1159.
87. Danser, A.H. and Deinum, J. (2005) Renin, prorenin and the putative (pro)renin receptor. *J. Renin Angiotensin Aldosterone Syst.* **6**, 163–165.
88. Nguyen, G., Delarue, F., Burckle, C., Bouzhir, L., Giller, T., and Sraer, J.D. (2002) Pivotal role of the renin/prorenin receptor in angiotensin II production and cellular responses to renin. *J. Clin. Invest.* **109**, 1417–1427.
89. Luetscher, J.A., Kraemer, F.B., and Wilson, D.M. (1989) Prorenin and vascular complications of diabetes. *Am. J. Hypertens.* **2**, 382–386.
90. Ichihara, A., Hayashi, M., Kaneshiro, Y., Suzuki, F., Nakagawa, T., Tada, Y., Koura, Y., Nishiyama, A., Okada, H., Uddin, M.N., et al. (2004) Inhibition of diabetic nephropathy by a decoy peptide corresponding to the "handle" region for nonproteolytic activation of prorenin. *J. Clin. Invest.* **114**, 1128–1135.

91. Ichihara, A., Kaneshiro, Y., and Suzuki, F. (2006) Prorenin receptor blockers: effects on cardiovascular complications of diabetes and hypertension. *Expert. Opin. Investig. Drugs* **15**, 1137–1139.
92. Sato, A., Hayashi, K., Naruse, M., and Saruta, T. (2003) Effectiveness of aldosterone blockade in patients with diabetic nephropathy. *Hypertension* **41**, 64–68.
93. Han, S.Y., Kim, C.H., Kim, H.S., Jee, Y.H., Song, H.K., Lee, M.H., Han, K.H., Kim, H.K., Kang, Y.S., Han, J.Y., et al. (2006) Spironolactone prevents diabetic nephropathy through an anti-inflammatory mechanism in type 2 diabetic rats. *J. Am. Soc. Nephrol.* **17**, 1362–1372.
94. Matsumoto, S., Takebayashi, K., and Aso, Y. (2006) The effect of spironolactone on circulating adipocytokines in patients with type 2 diabetes mellitus complicated by diabetic nephropathy. *Metabolism* **55**, 1645–1652.
95. van den Meiracker, A.H., Baggen, R.G., Pauli, S., Lindemans, A., Vulto, A.G., Poldermans, D., and Boomsma, F. (2006) Spironolactone in type 2 diabetic nephropathy: effects on proteinuria, blood pressure and renal function. *J. Hypertens.* **24**, 2285–2292.
96. Rossing, K., Schjoedt, K.J., Smidt, U.M., Boomsma, F., and Parving, H.H. (2005) Beneficial effects of adding spironolactone to recommended antihypertensive treatment in diabetic nephropathy: a randomized, double-masked, cross-over study. *Diabetes Care* **28**, 2106–2112.
97. Epstein, M., Williams, G.H., Weinberger, M., Lewin, A., Krause, S., Mukherjee, R., Patni, R., and Beckerman, B. (1998) Selective aldosterone blockade with eplerenone reduces albuminuria in patients with type 2 diabetes. *Clin. J. Am. Soc. Nephrol.* **1**, 940–951.
98. Ferrario, C.M. and Iyer, S.N. (1998) Angiotensin-(1-7): a bioactive fragment of the renin-angiotensin system. *Regul. Pept.* **78**, 13–18.
99. Trask, A.J. and Ferrario, C.M. (2007) Angiotensin-(1-7): pharmacology and new perspectives in cardiovascular treatments. *Cardiovasc. Drug Rev.* **25**, 162–174.
100. Hamming, I., Cooper, M.E., Haagmans, B.L., Hooper, N.M., Korstanje, R., Osterhaus, A.D., Timens, W., Turner, A.J., Navis, G., and van Goor, H. (2007) The emerging role of ACE2 in physiology and disease. *J. Pathol.* **212**, 1–11.
101. Santos, R.A., Simoes e Silva, A.C., Maric, C., Silva, D.M., Machado, R.P., de Buhr, I., Heringer-Walther, S., Pinheiro, S.V., Lopes, M.T., Bader, M., et al. (2003) Angiotensin-(1-7) is an endogenous ligand for the G protein-coupled receptor Mas. *Proc. Natl. Acad. Sci. U. S. A.* **100**, 8258–8263.
102. Ferrario, C.M. (1998) Angiotension-(1-7) and antihypertensive mechanisms. *J. Nephrol.* **11**, 278–283.
103. Burns, K.D. (2007) The emerging role of angiotensin-converting enzyme-2 in the kidney. *Curr. Opin. Nephrol. Hypertens.* **16**, 116–121.
104. Ronchi, F.A., Irigoyen, M.C., and Casarini, D.E. (2007) Association of somatic and N-domain angiotensin-converting enzymes from Wistar rat tissue with renal dysfunction in diabetes mellitus. *J. Renin Angiotensin Aldosterone Syst.* **8**, 34–41.
105. Ye, M., Wysocki, J., William, J., Soler, M.J., Cokic, I., and Batlle, D. (2006) Glomerular localization and expression of angiotensin-converting enzyme 2 and angiotensin-converting enzyme: implications for albuminuria in diabetes. *J. Am. Soc. Nephrol.* **17**, 3067–3075.
106. Lely, A.T., Hamming, I., van Goor, H., and Navis, G.J. (2004) Renal ACE2 expression in human kidney disease. *J. Pathol.* **204**, 587–593.
107. Wysocki, J., Ye, M., Soler, M.J., Gurley, S.B., Xiao, H.D., Bernstein, K.E., Coffman, T.M., Chen, S., and Batlle, D. (2006) ACE and ACE2 activity in diabetic mice. *Diabetes* **55**, 2132–2139.
108. Frojdo, S., Sjolind, L., Parkkonen, M., Makinen, V.P., Kilpikari, R., Pettersson-Fernholm, K., Forsblom, C., Fagerudd, J., Tikellis, C., Cooper, M.E., et al. (2005) Polymorphisms in the gene encoding angiotensin I converting enzyme 2 and diabetic nephropathy. *Diabetologia* **48**, 2278–2281.
109. Jessup, J.A., Gallagher, P.E., Averill, D.B., Brosnihan, K.B., Tallant, E.A., Chappell, M.C., and Ferrario, C.M. (2006) Effect of angiotensin II blockade on a new congenic model of hypertension derived from transgenic Ren-2 rats. *Am. J. Physiol. Heart Circ. Physiol.* **291**, H2166–H2172.
110. Ocaranza, M.P., Godoy, I., Jalil, J.E., Varas, M., Collantes, P., Pinto, M., Roman, M., Ramirez, C., Copaja, M., Diaz-Araya, G., et al. (2006) Enalapril attenuates downregulation of angiotensin-converting enzyme 2 in the late phase of ventricular dysfunction in myocardial infarcted rat. *Hypertension* **48**, 572–578.
111. Ruiz-Ortega, M., Esteban, V., and Egido, J. (2007) The regulation of the inflammatory response through nuclear factor-kappab pathway by angiotensin IV extends the role of the renin angiotensin system in cardiovascular diseases. *Trends Cardiovasc. Med.* **17**, 19–25.
112. Abrahamsen, C.T., Pullen, M.A., Schnackenberg, C.G., Grygielko, E.T., Edwards, R.M., Laping, N.J., and Brooks, D.P. (2002) Effects of angiotensins II and IV on blood pressure, renal function, and PAI-1 expression in the heart and kidney of the rat. *Pharmacology* **66**, 26–30.
113. Sugimoto, K.I., Fujimori, A., Yuyama, H., Tahara, A., and Fujimura, A. (2004) Renal protective effect of YM598, a selective endothelin type A receptor antagonist. *J. Cardiovasc. Pharmacol.* **44**, S451–S454.
114. Feldstein, C. and Romero, C. (2007) Role of endothelins in hypertension. *Am. J. Ther.* **14**, 147–153.
115. Granger, J.P., Abram, S., Stec, D., Chandler, D., and LaMarca, B. (2006) Endothelin, the kidney, and hypertension. *Curr. Hypertens. Rep.* **8**, 298–303.
116. Kohan, D.E. (2006) The renal medullary endothelin system in control of sodium and water excretion and systemic blood pressure. *Curr. Opin. Nephrol. Hypertens.* **15**, 34–40.
117. Neuhofer, W. and Pittrow, D. (2006) Role of endothelin and endothelin receptor antagonists in renal disease. *Eur. J. Clin. Invest.* **36(Suppl 3),** 78–88.
118. Sorokin, A. and Kohan, D.E. (2003) Physiology and pathology of endothelin-1 in renal mesangium. *Am. J. Physiol. Renal Physiol.* **285**, F579–F589.
119. Dean, R., Zhuo, J., Alcorn, D., Casley, D., and Mendelsohn, F.A.O. (1996) Cellular localization of endothelin receptor subtypes in the rat kidney following in vitro labelling. *Clin. Exp. Pharmacol. Phys.* **23**, 524–531.
120. Ding, S.S., Qiu, C., Hess, P., Xi, J.F., Zheng, N., and Clozel, M. (2003) Chronic endothelin receptor blockade prevents both early hyperfiltration and late overt diabetic nephropathy in the rat. *J. Cardiovasc. Pharmacol.* **42**, 48–54.
121. Hocher, B., Schwarz, A., Reinbacher, D., Jacobi, J., Lun, A., Priem, F., Bauer, C., Neumayer, H.H., and Raschack, M. (2001) Effects of endothelin receptor antagonists on the progression of diabetic nephropathy. *Nephron* **87**, 161–169.
122. Sasser, J.M., Sullivan, J.C., Hobbs, J.L., Yamamoto, T., Pollock, D.M., Carmines, P.K., and Pollock, J.S. (2007) Endothelin A receptor blockade reduces diabetic renal injury via an anti-inflammatory mechanism. *J. Am. Soc. Nephrol.* **18**, 143–154.
123. Pfab, T., Thone-Reineke, C., Theilig, F., Lange, I., Witt, H., Maser-Gluth, C., Bader, M., Stasch, J.P., Ruiz, P., Bachmann, S., et al. (2006) Diabetic endothelin B receptor-deficient rats develop severe hypertension and progressive renal failure. *J. Am. Soc. Nephrol.* **17**, 1082–1089.
124. Hargrove, G.M., Dufresne, J., Whiteside, C., Muruve, D.A., and Wong, N.C. (2000) Diabetes mellitus increases endothelin-1 gene transcription in rat kidney. *Kidney Int.* **58**, 1534–1545.
125. Glogowski, E.A., Tsiani, E., Zhou, X., Fantus, I.G., and Whiteside, C. (1999) High glucose alters the response of mesangial cell protein kinase C isoforms to endothelin-1. *Kidney Int.* **55**, 486–499.
126. Kohno, M., Horio, T., Ikeda, M., Yokokawa, K., Fukui, T., Yasunari, K., Kurihara, N., and Takeda, T. (1992) Angiotensin II stimulates endothelin-1 secretion in cultured rat mesangial cells. *Kidney Int.* **42**, 860–866.
127. Minchenko, A.G., Stevens, M.J., White, L., Abatan, O.I., Komjati, K., Pacher, P., Szabo, C., and Obrosova, I.G. (2003) Diabetes-induced overexpression of endothelin-1 and endothelin receptors in the rat renal cortex is mediated via poly(ADP-ribose) polymerase activation. *FASEB J.* **17**, 1514–1516.
128. Khan, M.A., Dashwood, M.R., Mumtaz, F.H., Thompson, C.S., Mikhailidis, D.P., and Morgan, R.J. (1999) Upregulation of endothelin A receptor sites in the rabbit diabetic kidney: potential relevance to the early pathogenesis of diabetic nephropathy. *Nephron* **83**, 261–267.
129. Tsiani, E., Lekas, P., Fantus, I.G., Dlugosz, J., and Whiteside, C. (2002) High glucose-enhanced activation of mesangial cell p38 MAPK by ET-1, ANG II, and platelet-derived growth factor. *Am. J. Physiol. Endocrinol. Metab.* **282**, E161–E169.
130. Hua, H., Goldberg, H.J., Fantus, I.G., and Whiteside, C.I. (2001) High glucose-enhanced mesangial cell extracellular signal-regulated protein kinase activation and alpha1(IV) collagen expression in response to endothelin-1: role of specific protein kinase C isozymes. *Diabetes* **50**, 2376–2383.
131. Hughes, A.K., Stricklett, P.K., Padilla, E., and Kohan, D.E. (1996) Effect of reactive oxygen species on endothelin-1 production by human mesangial cells. *Kidney Int.* **49**, 181–189.
132. Zhu, Y.C., Zhu, Y.Z., and Moore, P.K. (2006) The role of urotensin II in cardiovascular and renal physiology and diseases. *Br. J. Pharmacol.* **148**, 884–901.
133. Matsushita, M., Shichiri, M., Fukai, N., Ozawa, N., Yoshimoto, T., Takasu, N., and Hirata, Y. (2003) Urotensin II is an autocrine/paracrine growth factor for the porcine renal epithelial cell line, LLCPK1. *Endocrinology* **144**, 1825–1831.
134. Watanabe, T., Pakala, R., Katagiri, T., and Benedict, C.R. (2001) Synergistic effect of urotensin II with mildly oxidized LDL on DNA synthesis in vascular smooth muscle cells. *Circulation* **104**, 16–18.
135. Chen, Y., Zhao, M., Liu, X., Yao, W., Yang, J., Zhang, Z., and Tang, C. (2001) Urotensin II receptor in the rat airway smooth muscle and its effect on the rat airway smooth muscle cells proliferation. *Chin. Med. Sci. J.* **16**, 231–235.
136. Tzanidis, A., Hannan, R.D., Thomas, W.G., Onan, D., Autelitano, D.J., See, F., Kelly, D.J., Gilbert, R.E., and Krum, H. (2003) Direct actions of urotensin II on the heart: implications for cardiac fibrosis and hypertrophy. *Circ. Res.* **93**, 246–253.
137. Madeddu, P., Emanueli, C., and El-Dahr, S. (2007) Mechanisms of disease: the tissue kallikrein-kinin system in hypertension and vascular remodeling. *Nat. Clin. Pract. Nephrol.* **3**, 208–221.
138. Riad, A., Zhuo, J.L., Schultheiss, H.P., and Tschope, C. (2007) The role of the renal kallikrein-kinin system in diabetic nephropathy. *Curr. Opin. Nephrol. Hypertens.* **16**, 22–26.
139. Bhoola, K.D., Figueroa, C.D., and Worthy, K. (1992) Bioregulation of kinins: kallikreins, kininogens, and kininases. *Pharmacol. Rev.* **44**, 1–80.
140. Marceau, F. and Regoli, D. (2004) Bradykinin receptor ligands: therapeutic perspectives. *Nat. Rev. Drug Discov.* **3**, 845–852.
141. Ren, Y., Garvin, J., and Carretero, O.A. (2002) Mechanism involved in bradykinin-induced efferent arteriole dilation. *Kidney Int.* **62**, 544–549.
142. Zhang, J.C., Claffey, K., Sakthivel, R., Darzynkiewicz, Z., Shaw, D.E., Leal, J., Wang, Y.C., Lu, F.M., and McCrae, K.R. (2000) Two-chain high molecular weight kininogen induces endothelial cell apoptosis and inhibits angiogenesis:

partial activity within domain 5. *FASEB J.* **14**, 2589–2600.

143. Dean, R., Maric, C., Aldred, P., Zhuo, J., Casley, D., Muller-Esterl, W., Alcorn, D., and Mendelsohn, F.A.O. (1999) Rat renomedullary interstitial cells possess bradykinin type 2 receptors in vivo and in vitro. *Clin. Exp. Physiol. Pharmacol.* **26**, 48–55.
144. Kakoki, M., Takahashi, N., Jennette, J.C., and Smithies, O. (2004) Diabetic nephropathy is markedly enhanced in mice lacking the bradykinin B2 receptor. *Proc. Natl. Acad. Sci. U. S. A.* **101**, 13302–13305.
145. Tan, Y., Keum, J.S., Wang, B., McHenry, M.B., Lipstiz, S.R., and Jaffa, A.A. (2007) Targeted deletion of B2-kinin receptors protects against the development of diabetic nephropathy. *Am. J. Physiol. Renal Physiol.* **293(4),** F1026–F1035.
146. Campbell, D.J., Kelly, D.J., Wilkinson-Berka, J.L., Cooper, M.E., and Skinner, S.L. (1999) Increased bradykinin and "normal" angiotensin peptide levels in diabetic Sprague-Dawley and transgenic (mRen-2)27 rats. *Kidney Int.* **56**, 211–221.
147. Tschope, C., Schultheiss, H.P., and Walther, T. (2002) Multiple interactions between the renin-angiotensin and the kallikrein-kinin systems: role of ACE inhibition and AT1 receptor blockade. *J. Cardiovasc. Pharmacol.* **39**, 478–487.
148. Tikkanen, I., Tikkanen, T., Cao, Z., Allen, T.J., Davis, B.J., Lassila, M., Casley, D., Johnston, C.I., Burrell, L.M., and Cooper, M.E. (2002) Combined inhibition of neutral endopeptidase with angiotensin converting enzyme or endothelin converting enzyme in experimental diabetes. *J. Hypertens.* **20**, 707–714.
149. Davis, B.J., Johnston, C.I., Burrell, L.M., Burns, W.C., Kubota, E., Cao, Z., Cooper, M.E., and Allen, T.J. (2003) Renoprotective effects of vasopeptidase inhibition in an experimental model of diabetic nephropathy. *Diabetologia* **46**, 961–971.
150. Zaoui, P., Cantin, J.F., Alimardani-Bessette, M., Monier, F., Halimi, S., Morel, F., and Cordonnier, D. (2000) Role of metalloproteases and inhibitors in the occurrence and progression of diabetic renal lesions. *Diabetes Metab.* **26(Suppl 4),** 25–29.
